# The effectiveness of applying a competency-oriented mentorship system to clinical microbiology practice teaching

**DOI:** 10.3389/fmed.2026.1954876

**Published:** 2026-10-29

**Authors:** Di Mu, Menghao Wang, Wenfeng Zhang

**Affiliations:** 1 Department of Laboratory Medicine, The Second Affiliated Hospital of Chongqing Medical University, Chongqing, China; 2 Department of Hepatobiliary Surgery, The Second Affiliated Hospital of Chongqing Medical University, Chongqing, China

**Keywords:** clinical practice, mentorship system, microbiology testing, post competency, traditional apprenticeship

## Abstract

**Objective:**

Existing studies lack a competency-oriented integration of multiple teaching strategies within a mentorship system. This study applies such an integration to clinical microbiology internships to inform teaching reform.

**Methods:**

Forty undergraduate interns who practiced clinical microbiology in the laboratory department of a hospital from July 2023 to May 2024 were selected and randomly divided into an experimental group and a control group. The control group received a traditional apprenticeship model, while the experimental group followed a competency-oriented mentorship system. After the internship, the theoretical assessment, practical skills assessment scores, self-evaluation scores of post competency, and teaching satisfaction were compared between the two groups.

**Results:**

The theoretical scores and practical skills scores of the experimental group were significantly higher than those of the control group. In the self-evaluation of post competency, the experimental group also scored significantly higher in dimensions such as clinical thinking ability, and communication and collaboration skills. The overall satisfaction with the teaching model in the experimental group was significantly higher than that in the control group.

**Conclusion:**

The competency-oriented mentorship system teaching model can effectively enhance the theoretical knowledge, operational skills, and comprehensive professional competence of medical laboratory interns in the field of microbiology, with significantly better teaching outcomes than the traditional apprenticeship model.

## Introduction

Clinical microbiology testing represents a key branch of medical laboratory science. It directly influences the diagnosis of infectious diseases, the rational use of antibiotics, and the control of hospital infections (1, 2). As global public health now faces dual threats from emerging and re-emerging infectious diseases as well as bacterial resistance, clinical microbiology professionals are expected to meet higher standards of competence (3). The advancement of modern laboratory medicine calls for practitioners who not only possess the technical skills required for automated testing processes, but also demonstrate the ability to clinically interpret complex results, collaborate effectively with clinicians, handle emergencies under multiple pressures, and pursue scientific innovation to drive the field forward (4, 5). This renewed emphasis on “skills” fundamentally reflects the core meaning of “job competence.”

However, the traditional internship teaching model for medical laboratories in China largely originates from the “master-apprentice” approach to skill transmission. This model tends to prioritize hands-on operations, standardized procedures, and rote imitation during practice, while neglecting the development of integrated clinical thinking and scientific innovation (6). In this relatively passive and fragmented learning environment, interns find it difficult to connect isolated testing procedures with the complete clinical diagnostic process. This difficulty limits the growth of their clinical thinking and hinders effective training in proactively identifying and solving problems. As a result, a noticeable gap emerges between their current skills and the actual requirements of future clinical practice (7). Competency-based medical education (CBME) emphasizes outcome-based, learner-centered training and integrated assessment of knowledge, skills, and professional behaviors rather than isolated technical procedures (8–12). Assessment in CBME should therefore capture clinical reasoning, communication, collaboration, and research capacity. Such a training model contradicts the competency-based, job-oriented medical education reform concept promoted by China's New Medical Education initiative.

To address this issue, researchers have explored and applied various teaching models in clinical laboratory medicine (13–15). One such model is the mentorship system, an individualized training approach that originated at Oxford University. This system has demonstrated benefits across multiple areas of medical education by fostering close teacher-student relationships and providing students with thorough, in-depth guidance (16, 17). International mentorship literature from Europe and North America has shown that mentorship can support clinical competence, professional identity formation, and research engagement (18–20). Previous studies indicate that the mentorship system plays a positive role in improving undergraduate interns' clinical competence and in shaping their professional qualities (21, 22). Recently, the mentorship system has continued to evolve, moving from a single-mentor model to mentor team systems, dual-mentor arrangements, and other forms. These changes aim to integrate multidisciplinary strengths and offer students a more comprehensive knowledge framework (17, 23). However, most existing studies focus on the effects of single interventions, and few have carried out comprehensive research that combines multiple teaching strategies under a job competency framework while building on the mentorship system. Consequently, it remains unknown how to organically integrate the training goal of “job competency” with the mentorship system and apply this integration to the highly specialized clinical microbiological testing internship. Therefore, the present study takes a job competency-oriented approach and uses the mentorship system as a support, with the aim of developing a new teaching method suitable for the clinical microbiological testing internship. This work is intended to offer practical insights for deepening teaching reform in medical laboratory internships.

## Research subjects and methods

### Research subjects

This was a single-center, exploratory, randomized teaching study. Forty eligible undergraduate interns majoring in medical laboratory technology who underwent a two-month internship in the microbiology section of the clinical laboratory department of our hospital between July 2023 and May 2024 were enrolled during the fixed rotation period as study participants. They were stratified by their scores and randomly divided into a control group and an experimental group, with 20 individuals in each group. There were no statistically significant differences in age, sex ratio, or baseline scores between the two groups. All research subjects gave informed consent and signed the informed consent forms.

### Teaching content and evaluation

Teaching content followed the internship syllabus and rotation requirements of the College of Laboratory Medicine of Chongqing Medical University. Eight laboratory physicians/technologists with intermediate or senior professional titles, each having more than five years of experience in clinical microbiology work and teaching, instructors (for the control group) or supervising mentors (for the experimental group). All received unified training before the study was carried out.

### Control group

A traditional apprenticeship model was used. After joining the microbiology section, students rotated according to the internship syllabus. The instructor explained and demonstrated the day's tasks, and students mainly watched and copied what the instructor did. They also attended a weekly mini-lecture organized by the department.

### Experimental group

On top of the traditional model, a competency-oriented mentorship model was implemented. Before starting, each mentor understood clearly the competency training goals and their own duties. Each mentor took five students, and within the first week of the rotation, the mentor interviewed and assessed each student to make an individualized internship plan, setting goals for professional knowledge, hands-on skills, clinical reasoning, communication and teamwork, and research ability. The teaching process included: (1) The mentor systematically explained and demonstrated the whole workflow from sample receipt to report review, with emphasis on aseptic technique, smear staining, isolation and culture, identification and susceptibility testing, and result reading. (2) The mentor selected typical clinical cases (bloodstream infection, respiratory infection, etc.) to guide students through analyzing test requests, interpreting results, and making differential diagnoses using clinical data. Weekly case discussions were held to develop students' clinical reasoning and communication skills. (3) Drawing on their own research direction or ongoing projects in the microbiology section, the mentor guided students to search and read literature, learn recent advances in microbiology, and complete either a literature presentation or part of a small research project (data collection and analysis, etc.), helping students build basic research thinking.

### Outcome measures and evaluation methods

All outcome measures were assessed immediately at the end of the 2-month internship. No long-term follow-up was performed. Theory and skill exams: At the end of the internship, all students took a closed-book exam (full score 100), covering basic microbiology theory, identification procedures for common pathogens, interpretation of susceptibility results, and clinical significance. Each student independently performed a “Gram stain and microscopic examination report on a sputum sample” which was scored by two senior faculty members not involved in the teaching, using a standard scoring rubric (full score 100).

A self-assessment questionnaire on job competency was used, covering five areas: clinical reasoning, communication and teamwork, research and innovation ability, basic professional knowledge, and practical skills. The questionnaire had 20 items, each rated on a 5-point Likert scale (1 = strongly disagree, 5 = strongly agree). The average score was then calculated for each dimension. The job competency questionnaire was self-designed for this exploratory study and has not undergone formal psychometric validation. It was used only as an internal self-assessment tool.

A self-designed questionnaire was used to survey all students' overall satisfaction with the teaching model. Response options were “satisfied,” “neutral,” or “dissatisfied.” Total satisfaction rate = (number satisfied + number neutral)/total number  ×  100%. Figure 1 presents the instructional procedures for both groups.

**Figure 1:**
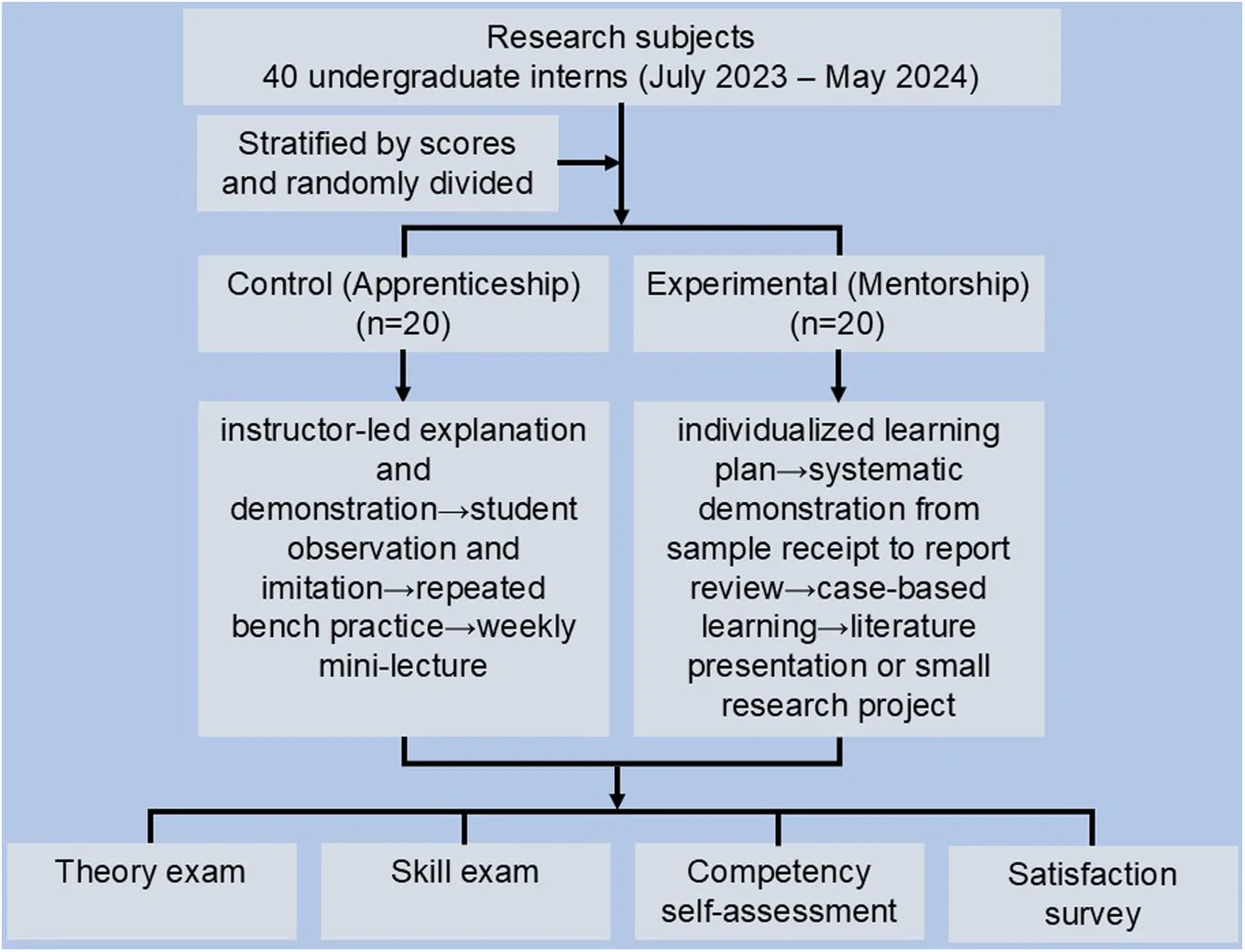
Comparison of instructional procedures in the control and experimental groups. Both groups completed the same rotation syllabus and end-of-rotation assessments.

### Statistical analysis

Data were analyzed using SPSS18.0. Continuous variables are expressed as mean ± standard deviation (x ± s). Between-group mean differences with 95% confidence intervals (95% CI) and Cohen's d were calculated. Categorical variables are expressed as counts and percentages, and the chi-square test was used for between-group comparisons. A *p*-value < 0.05 was considered statistically significant.

## Results

### Results of the theory and skill exams

After the internship ended, the theory exam scores and skill operation scores of students in the experimental group were significantly higher than those in the control group (Table 1).

**Table 1:** Results of the theory and skill exams.

Evaluation items	Experimental group (*n* = 20)	Control group (*n* = 20)	Mean difference (95% CI)	Cohen's d	*p* value
Theory exam	89.70 ± 2.27	80.55 ± 2.54	9.15 (7.61–10.69)	3.80	0.000*
Skill operation	91.60 ± 2.32	83.40 ± 3.05	8.20 (6.47–9.93)	3.03	0.000*

*, a *p* < 0.05 indicates a statistically significant difference.

### Results of self-evaluation results of job competency

In the experimental group, students' self-evaluation scores for clinical reasoning, communication and collaboration, and research and innovation ability were significantly higher than those in the control group (Table 2). For basic professional knowledge and practical operation ability, although students in the experimental group scored higher, the differences compared to the control group were not statistically significant (Table 2).

**Table 2:** Results of self-evaluation results of job competency.

Evaluation items	Experimental group (*n* = 20)	Control group (*n* = 20)	Mean difference (95% CI)	Cohen's d	*p* value
Clinical reasoning	4.58 ± 0.36	3.86 ± 0.43	0.71 (0.46–0.96)	1.82	0.000*
Communication and collaboration	4.44 ± 0.31	3.58 ± 0.36	0.86 (0.65–1.08)	2.56	0.000*
Research and innovation ability	4.41 ± 0.27	3.44 ± 0.33	0.98 (0.78–1.17)	3.22	0.000*
Basic professional knowledge	4.55 ± 0.31	4.38 ± 0.29	0.18 (−0.02–0.37)	0.57	0.072
Practical operation ability	4.30 ± 0.25	4.18 ± 0.30	0.11 (−0.07–0.29)	0.44	0.208

*, a *p* < 0.05 indicates a statistically significant difference.

### Results of the teaching satisfaction survey

The questionnaire results showed that the overall satisfaction rate with the teaching model was 95% in the experimental group, which was significantly higher than 60% in the control group, and the difference was statistically significant (Table 3).

**Table 3:** Results of the teaching satisfaction survey.

Evaluation items	Experimental group	Control group	OR	Cohen's h	*p* value
Number of cases	20	20	/	/	/
Fatisfaction	16（80%）	8（40%）	/	/	/
Fair	3（15%）	4（20%）	/	/	/
Dissatisfaction	1（5%）	8（40%）	/	/	/
Overall satisfaction	19（95%）	12（60%）	12.67	0.92	0.02*

OR, odds ratio. *, a *p* < 0.05 indicates a statistically significant difference.

## Discussion

The results of this study show that implementing a competency-oriented mentorship model in clinical microbiology internships achieved good outcomes. First, in terms of knowledge and skill acquisition, the experimental group outperformed the control group on both the theory and skill exams. This may be due to the more systematic and individualized guidance that mentorship offers (24). Unlike the traditional apprenticeship model, where students passively watch and repeat standardized operations, the mentor, based on the student's level of understanding and learning pace, could target weak areas and, through repeated practice and feedback, help the student gain a firmer grasp of the core knowledge and key skills in microbiology testing (16). The apprenticeship model often treats all learners the same, whereas the mentorship model adjusts teaching strategies to individual needs, leading to more efficient skill acquisition.

In terms of comprehensive ability development, the experimental group also showed significant improvement in self-rated scores for clinical reasoning, communication and collaboration, and research and innovation ability. This is closely related to the introduction of case-based teaching. By analyzing real clinical cases, students learned to combine fragmented test data with patient clinical information to make comprehensive judgments, effectively bridging the gap between the laboratory and the clinic (25). Notably, the self-evaluation scores for basic professional knowledge and practical operation ability, though higher in the experimental group, did not reach statistical significance. One possible explanation is that the traditional apprenticeship model, despite its limitations, still provides sufficient hands-on practice for routine technical skills, so the advantage of mentorship in these areas may take longer to manifest or may require more sensitive assessment tools. The research initiation and extracurricular activities guided by mentors stimulated students' spirit of exploration, helping them gain basic skills in literature searching, study design, and data analysis, which laid a solid foundation for their future career development. These activities are typically absent in the apprenticeship model, where students focus almost exclusively on bench work. Our results are consistent with previously reported findings (26–28). These findings are consistent with international evidence on mentorship and competency-based training (8, 18–20). Meanwhile, the higher satisfaction rate in the experimental group further indicates that this teaching model is well received by students. A mentor is not only a transmitter of knowledge but also a guide on students' professional path (29). This close mentor-student interaction enhanced students' learning experience and sense of belonging, and mobilized their initiative (30). In contrast, the distant, one-to-many relationship in the traditional apprenticeship model often leaves students feeling unsupported in their individual learning needs.

Although this study achieved positive results, some limitations remain. First, this single-center study with 40 participants limits generalizability. Multi-center studies with larger and more diverse samples are needed before the model can be widely recommended. Second, the short-term evaluation does not show whether these gains persist or translate into workplace performance. Longitudinal tracking of graduates' career competence, clinical performance, and professional development is recommended. Third, the competency outcomes relied on self-assessment, which may introduce response bias. Future studies should incorporate objective measures such as supervisor ratings, direct observation, or OSCE-style assessments. The sample size was limited and no *a priori* power calculation was performed; thus, the results should be interpreted as exploratory. In addition, the self-designed competency questionnaire lacks formal psychometric validation. This limits reproducibility and comparability across settings. Formal validation is essential before the questionnaire is used in future studies.

Wider adoption of this mentorship model would depend on institutional support. Mentors would need training in competency-based supervision, feedback, and assessment. The model also requires protected teaching time, recognition of mentoring workload, and a manageable mentor-to-student ratio. In our study, the ratio was approximately 1:5. Mentor teams, shared case libraries, digital tools, and standardized assessment rubrics could help make the model more scalable. Institutions with limited resources may still struggle to recruit enough mentors or adjust workloads. Future multi-center studies should examine cost-effectiveness and resource needs, and whether the model has a lasting effect on graduates' competence.

In summary, this study confirms that a competency-oriented mentorship model can significantly improve medical laboratory interns' theoretical knowledge, practical skills, and overall professional abilities in clinical microbiology—including clinical reasoning, communication and collaboration, and research innovation. Both teaching outcomes and student satisfaction were superior to those of the traditional apprenticeship model. This approach offers an effective teaching pathway for cultivating high-quality, innovative medical laboratory professionals and holds potential for wider application in clinical internship education.

## Data Availability

The original contributions presented in the study are included in the article/Supplementary Material, further inquiries can be directed to the corresponding author.

## References

[1] Dunbar SA Benavides M Gardner C. Advances in diagnostic methods for detection of bloodstream pathogens and antibiotic resistance determinants. Front Cell Infect Microbiol. (2025) 15:1725920–551. 10.3389/fcimb.2025.1725920 41446279 PMC12722983

[2] Zakhour J Haddad SF Kerbage A Wertheim H Tattevin P Voss A, International society of antimicrobial chemotherapy (ISAC) and the alliance for the prudent use of antibiotics (APUA), et al.. Diagnostic stewardship in infectious diseases: a continuum of antimicrobial stewardship in the fight against antimicrobial resistance. Int J Antimicrob Agents. (2023) 62:106816–1471. 10.1016/j.ijantimicag.2023.106816 37061101

[3] Salmanton-García J Maio G Stahl A De Barra JP Jensen-Fangel E Torti S. Specialist training in infectious diseases in Europe. Clin Microbiol Infect. (2025) 31:1003–10. 10.1016/j.cmi.2024.12.026 39725078

[4] Plebani M. Advancing value-based laboratory medicine. Clin Chem Lab Med. (2025) 63:249–57. 10.1515/cclm-2024-0823 39072502

[5] Lippi G Ognibene A. Perspectives on emerging challenges and training needs for the laboratory medicine specialist of the future. Adv Lab Med. (2026) 7:16–25. 10.1515/almed-2025-0146 41853782 PMC12994699

[6] Kim DH Kim J Park DH Lee JH Choi KD Lee GH. From technique mastery to scientific thinking: how open-ended experiments shape medical students’ perception of laboratory course roles in China. BMC Med Educ. (2024) 24:1488–452. 10.1186/s12909-024-06325-x 39696313 PMC11657453

[7] Yang Y Xu S Chen B Chen S Xu W Lu L. Educational strategies for enhancing medical students’ competency in laboratory medicine practice: a scoping review. Front Med (Lausanne). (2026) 13:1799809–718. 10.3389/fmed.2026.1799809 42200058 PMC13199127

[8] Frank JR Snell LS Cate OT Holmboe ES Carraccio C Swing SR. Competency-based medical education: theory to practice. Med Teach. (2010) 32(8):638–45. 10.3109/0142159X.2010.501190 20662574

[9] Li B Huang S Liao S He M Xiao Y Zhong E. Modified endoscopic purse-string suture with dental floss traction in the management of a duodenal defect. Acad Med. (2002) 77:361–7. 10.1055/a-2590-8450 40419268 PMC12105928

[10] Ten Cate O Scheele F. Competency-based postgraduate training: can we bridge the gap between theory and clinical practice? Acad Med. (2007) 82:542–7. 10.1097/ACM.0b013e31805559c7 17525536

[11] Holmboe ES Sherbino J Long DM Swing SR Frank JR. The role of assessment in competency-based medical education. Med Teach. (2010) 32:676–82. 10.3109/0142159X.2010.500704 20662580

[12] Englander R Cameron T Ballard AJ Dodge J Bull J Aschenbrener CA. Toward a common taxonomy of competency domains for the health professions and competencies for physicians. Acad Med. (2013) 88:1088–94. 10.1097/ACM.0b013e31829a3b2b 23807109

[13] Li H Sun J Zhou Y Ding S Guo Y Jiang Q. The utility of competency-oriented clinical laboratory teaching combined with case-based learning (CBL). Clin Chem Lab Med. (2021) 59:1784–9. 10.1515/cclm-2021-0467 34225395

[14] Turkel S Ramirez-Vasquez M Abdi J Nasr P. Evaluating the impact of remote teaching on competency based outcomes in clinical laboratory education. Am J Clin Pathol. (2026) 165(2):aqaf155. 10.1093/ajcp/aqaf155 41698406

[15] Feng X Wu W Bi Q. Reform of teaching and practice of the integrated teaching method BOPPPS-PBL in the course “clinical haematological test technique”. BMC Med Educ. (2024) 24:773. 10.1186/s12909-024-05765-9 39030580 PMC11264887

[16] Ong D Zapf TC Cevik M Palacios-Baena ZR Barać A Cimen C. Current mentorship practices in the training of the next generation of clinical microbiology and infectious disease specialists: an international cross-sectional survey. Eur J Clin Microbiol Infect Dis. (2019) 38:659–65. 10.1007/s10096-019-03509-y 30783890 PMC6424943

[17] Teo MYK Ibrahim H Lin CKR Hamid NABA Govindasamy R Somasundaram N. Mentoring as a complex adaptive system - a systematic scoping review of prevailing mentoring theories in medical education. BMC Med Educ. (2024) 24:726. 10.1186/s12909-024-05707-5 38970020 PMC11225364

[18] Sambunjak D Straus SE Marušić A. Mentoring in academic medicine: a systematic review. JAMA. (2006) 296:1103–15. 10.1001/jama.296.9.1103 16954490

[19] Frei E Stamm M Buddeberg-Fischer B. Mentoring programs for medical students-a review of the PubMed literature 2000–2008. BMC Med Educ. (2010) 10:32. 10.1186/1472-6920-10-32 20433727 PMC2881011

[20] Kashiwagi DT Varkey P Cook DA. Mentoring programs for physicians in academic medicine: a systematic review. Acad Med. (2013) 88:1029–37. 10.1097/ACM.0b013e318294f368 23702518

[21] Abdelmannan D Buhumaid R Salman H Ba Madhaf W AlRajaby H Zary N. A scoping review of mentorship in graduate medical education: a proposed conceptual framework. Front Med (Lausanne). (2025) 12:1616148. 10.3389/fmed.2025.1616148 40901506 PMC12400154

[22] Ventimiglia G Setti I Maffoni M. Mentoring in hospital settings: a systematic review of guidance, care, and professional development. Healthcare (Basel). (2026) 14(4):505. 10.3390/healthcare14040505 41754018 PMC12941059

[23] Moore-Lotridge SN Conover GM Finck LR Schoenecker JG Hu PJ Eley DS. Strength in numbers: leveraging mentorship teams to support medical student research in turbulent research environments. Med Sci Educ. (2026) 36:161–71. 10.1007/s40670-025-02575-6 41939020 PMC13043834

[24] Sedigh A Bagheri S Naeimi P Rahmanian V Sharifi N. The effect of peer mentoring program on clinical academic progress and psychological characteristics of operating room students: a parallel randomized controlled trial. BMC Med Educ. (2024) 24:438. 10.1186/s12909-024-05424-z 38649841 PMC11036741

[25] Wang QW Zhang JJ Zhang H Wang AH Wu YY Duan Z. Bridging the theory-practice gap in laboratory medicine: development and application of a standardized case library for fostering clinical reasoning in postgraduate training. Front Med. (2026) 13:1834799. 10.3389/fmed.2026.1834799 PMC13342040 42422848

[26] Zhou K Cao C Liu X Sun M Wu Z Zheng W. The impact of exposure to scientific research and inclusive mentoring style on medical undergraduates’ perceptions of critical thinking, communication, and passion. Med Educ Online. (2025) 30:2535406. 10.1080/10872981.2025.2535406 40726104 PMC12308871

[27] Lee M Hu W Bilszta J. Determining expected research skills of medical students on graduation: a systematic review. Med Sci Educ. (2020) 30:1465–79. 10.1007/s40670-020-01059-z 34457814 PMC8368133

[28] Zerzan JT Hess R Schur E Phillips RS Rigotti N. Making the most of mentors: a guide for mentees. Acad Med. (2009) 84:140–4. 10.1097/ACM.0b013e3181906e8f 19116494

[29] Ehsanian V Derijani F Khorraminia M Eiliaei S. Implementation and evaluation of a mentoring and counseling program for medical students at shahid beheshti university of medical sciences. BMC Med Educ. (2025) 25:940. 10.1186/s12909-025-07488-x 40598190 PMC12211925

[30] Tam G Wong NS Lee SS. A survey of the involvement of primary care doctors in HIV prevention and care in a low-prevalence, high-income setting. BMC Fam Pract. (2021) 22:27. 10.1186/s12875-021-01376-1 33504326 PMC7842046

